# Psychrophiles to control ice-water phase changes in frost-susceptible soils

**DOI:** 10.1038/s41598-023-51060-w

**Published:** 2024-01-04

**Authors:** Rashed Rahman, Tejo V. Bheemasetti, Tanvi Govil, Rajesh Sani

**Affiliations:** 1 Department of Civil and Architectural and Engineering Mechanics, University of Arizona, Tucson, AZ 85721 USA; 2 Department of Chemical and Biological Engineering, South Dakota School of Mines and Technology, Rapid City, SD 57701 USA

**Keywords:** Civil engineering, Applied microbiology

## Abstract

The phase changes of soil water or porous media have a crucial influence on the performance of natural and man-made infrastructures in cold regions. While various methods have been explored to address the impacts of frost-action arising from these phase changes, conventional approaches often rely on chemicals, mechanical techniques, and the reuse of waste materials, which often exhibit certain limitations and environmental concerns. In contrast, certain organisms produce ice-binding proteins (IBPs) or antifreeze proteins (AFPs) to adapt to low temperatures, which can inhibit ice crystal growth by lowering the freezing point and preventing ice crystallization without the need for external intervention. This study explores the potential of three psychrophilic microbes: *Sporosarcina psychrophile*, *Sporosarcina globispora*, and *Polaromonas hydrogenivorans*, to induce non-equilibrium freezing point depression and thermal hysteresis in order to control ice lens growth in frost-susceptible soils. We hypothesize that the AFPs produced by psychrophiles will alter the phase changes of porous media in frost-susceptible soils. The growth profiles of the microbes, the concentration of released proteins in the extracellular solution, and the thermal properties of the protein-mixed soils are monitored at an interval of three days. The controlled soil showed a freezing point of − 4.59 °C and thermal hysteresis of 4.62 °C, whereas protein-treated soil showed a maximum freezing point depression of − 8.54 °C and thermal hysteresis of 7.71 °C. Interestingly, except for the controlled sample, all the protein-treated soil samples were thawed at a negative temperature (minimum recorded at − 0.85 °C). Further analysis showed that the treated soils compared to porous media mixed soil freeze (1.25 °C vs. 0.51 °C) and thaw (2.75 °C vs. 1.72 °C) at extensive temperature gap. This freezing and thawing temperature gap is the temperature difference between the beginning of ice core formation and completed frozen, and the beginning of ice core thawing and completed thawed for the treated soil samples selected from different incubation days. Overall, this study presents a novel bio-mediated approach using psychrophilic microbes to control ice formation in frost-susceptible soils.

## Introduction

In the cold regions, soil water, also referred to as porous media, plays a critical role in governing frost-heave and thaw-weakening induced distresses to the natural and built-in infrastructures^1^. Frost-action, a seasonal repetition constituting cyclic freezing and thawing phenomenon has an enduring impact on the engineering properties of soil^2,3^. Though the frost-action is controlled by freezing temperature, other factors like the availability of water, frost-susceptible soil, and the pore network play a pivotal role in the ice nucleation and crystallization in soil for compelling frost-action^4–6^. Taber (1929) experimentally studied these factors to understand water migration towards a growing ice lens and provided a distinction between frozen soil, frozen fringe, and unfrozen soil layers^7^. Following Taber and many subsequent studies over the last nine decades have focused on understanding and controlling the growth of ice lenses in porous media. Stabilization treatment techniques like cement, fly ash, inorganic salts, and structural polymer foam injection (e.g. Uretek 486 STAR) have been tested to arrest the growth of ice lenses either by controlling drainage or by chemically lowering the freezing point of the soil-porous media^8–11^. Though they are effective case by case, the resiliency and the environmental concerns have prompted engineers to find a better solution^12–14^. As an alternative to current approaches, a novel nature-based bio-mediated approach to mitigate and control the ice formation of porous media has been presented in this study.

In nature, under subfreezing conditions, eukaryotes (e.g., plants, insects, fishes), and prokaryotes (e.g., bacteria, archaea) have evolved to adapt and overcome the effects of low temperatures via the secretion and production of a diverse class of proteins collectively called ice-binding proteins (IBPs)^15^. IBPs are known to be particularly efficient in binding and active on the interface between ice and liquid water. This characteristic allows them to modulate the growth of ice crystals in multiple ways, including suppressing the freezing point of water, inhibiting ice recrystallization, shaping ice crystals, and controlling ice nucleation^15^. Antifreeze proteins (AFPs) are proteins in the family of low temperature associated IBP’s that are also known to decrease the freezing point by enacting thermal hysteresis (TH), i.e., by reducing the freezing point of water while also increasing the melting temperature of ice crystals^16^. Consequently, liquid water does not entirely freeze if it holds dissolved AFPs, even at temperatures lower than the melting point of ice.

Chasnitsky and Braslavsky, 2019, explained the concept behind AFP’s freezing repression mechanism through the Gibbs–Thompson phenomenon. Herein, the authors explain that the AFP’s adsorbed to the surface of the ice crystals limits the growth of ice between the proteins and develop locally significant convex surface regions. These interfacial curvatures are energetically less favorable for water molecules to join the ice lattice and the high surface curvature reduces the melting point, agreeing with the Gibbs–Thomson (Kelvin) effect^17^. Simultaneously, AFPs tend to diffuse the Water–Ice interface to Water-AFP-Ice interfacial region due to the drop in Gibbs energy. The AFP concentration in the Water-AFP-Ice interfacial region leads to the changes in freezing point depression due to local colligative impact^17^. This presupposition of action of AFPs is, therefore, considered dependent on (a) the face of ice crystal planes an AFP can bind to; (b) the factors influencing the stability of AFP binding to these ice crystal planes, (c) the concentration of AFP proteins and the different capacities of different types of antifreeze proteins to cause thermal hysteresis at equimolar concentrations^18^. Recently, it was suggested that the AFPs’ functionality can also prevent ice formation by binding to living hosts’ cell surfaces, where the ice nuclei can form, and via this mechanism, AFPs can protect cells from the hypothermic cold shock (preceding the emergence of ice) damage^19^.

Thus, emphasizing that “ice-binding” (or “antifreeze”) proteins can fundamentally work with different mechanisms, it is worth bringing or mentioning another category of peptides or proteins termed cold shock proteins (CSPs), as well as the cold acclimation proteins (CAPs), that characteristically enable psychrophilic microbes adapted to cope with the hypothermal stress due to abrupt temperature drop^20–22^. While the production of CSPs is transient and increases as a rapid overexpression with the severity of the cold, CAPs are continuously synthesized during prolonged growth at low temperatures (5–10 °C in psychrotolerants, and 0–5 °C in psychrophiles)^23^. CSPs and CAPs have been validated to help cells counteract some harmful effects of temperature downshift by balancing negative consequences like low germination, cell damage, etc.^24,25^. Interestingly, cold conditions are not the only source of inducement reported for CSPs are suggested to be induced also to promote the viability of the hosts under broader stress responses and, for example, under nutritional upshifts^26^. Functions of non-cold-inducible CSPs are, however, poorly understood^27^.

In terms of inducibility, compared to their mesophilic counterparts, CSPs or CAPs proteins in psychrophiles are more consistently expressed instead of being transiently induced during the cold acclimation phase. Cold-adapted enzymes from psychrophiles have high specific activities at low temperatures, often up to ten orders of magnitude to compensate for the slow reaction rates at low temperatures, compared to their mesophilic counterparts^28^. These instances suggest that CSPs or CAPs play more significant roles in survival in cold environments, including extremely cold polar climates for psychrophiles, than their mesophilic counterparts^29^. On a similar note, different microbes possess their own family of CSPs or CAPs homologs. Although CSP genes have been identified in psychrophiles’ genomes, their contributions to freeze–thaw adaptations have been elucidated only in few studies^30^. There is still scantiness of quantitative numbers about the cold-shock response by cold-adapted proteins in cold-adapted bacteria below freezing temperatures. To our knowledge, no research has studied or explicated the characteristic of CSPs to arrest ice crystallization and the potential to show thermal hysteresis (TH).

It has been proposed that the lower temperature limit for psychrophiles can go down to − 12 °C for reproduction and − 20 °C for their metabolic functions^31^. Hence, through this manuscript, we emphasize the hypothesis that even those psychrophilic microbes that do not have AFPs in their genome are capable of hysteresis, i.e., changing the freezing/melting curve of water/ice. Besides the fundamental research importance in biological sciences, identifying and exploring new unique or novel hysteresis proteins or active biomolecules that can inhibit the growth and recrystallization of ice in non-AFP producing psychrophiles is of broad interest in biomedicine, aquaculture, agriculture, frozen food industries, and organ preservation.

Drawing upon existing literature, our hypothesis posits that Anti-Freeze Proteins (AFPs) derived from psychrophilic organisms can serve as effective agents for controlling the formation of ice lenses within pore fluids at soil interfaces. This study aims to delve into and present compelling evidence regarding the potential of psychrophilic strains such as *Sporosarcina psychrophile*, *Sporosarcina globispora*, and *Polaromonas hydrogenivorans*. Specifically, we seek to showcase their ability to induce non-equilibrium freezing point depression and thermal hysteresis (TH), ultimately exerting control over the growth of ice lenses in frost-susceptible soils. In the field of geotechnical research, laboratory-based equipment are extensively used to determine the soil thermal properties like freezing and thawing point, thermal hysteresis, thermal conductivity, specific heat capacity, and measurement of unfrozen water content^32–35^. While advancements in numerical simulations and lab-built modeling have expanded our understanding, the direct observation of phase changes has remained constrained^36,37^. In this study, a thermoelectrically temperature-controlled cooling device is used to measure the freezing and thawing points^38^. Notably, we integrate a microscopic setup with the cooling system, allowing real-time observation of phase changes in porous media during experiments. This approach provides a unique advantage over traditional methods like differential scanning calorimetry, offering direct visualization of transformations within porous media.

## Results

### Growth and protein profiles of the three psychrophilic microbes

**Figure 1:**
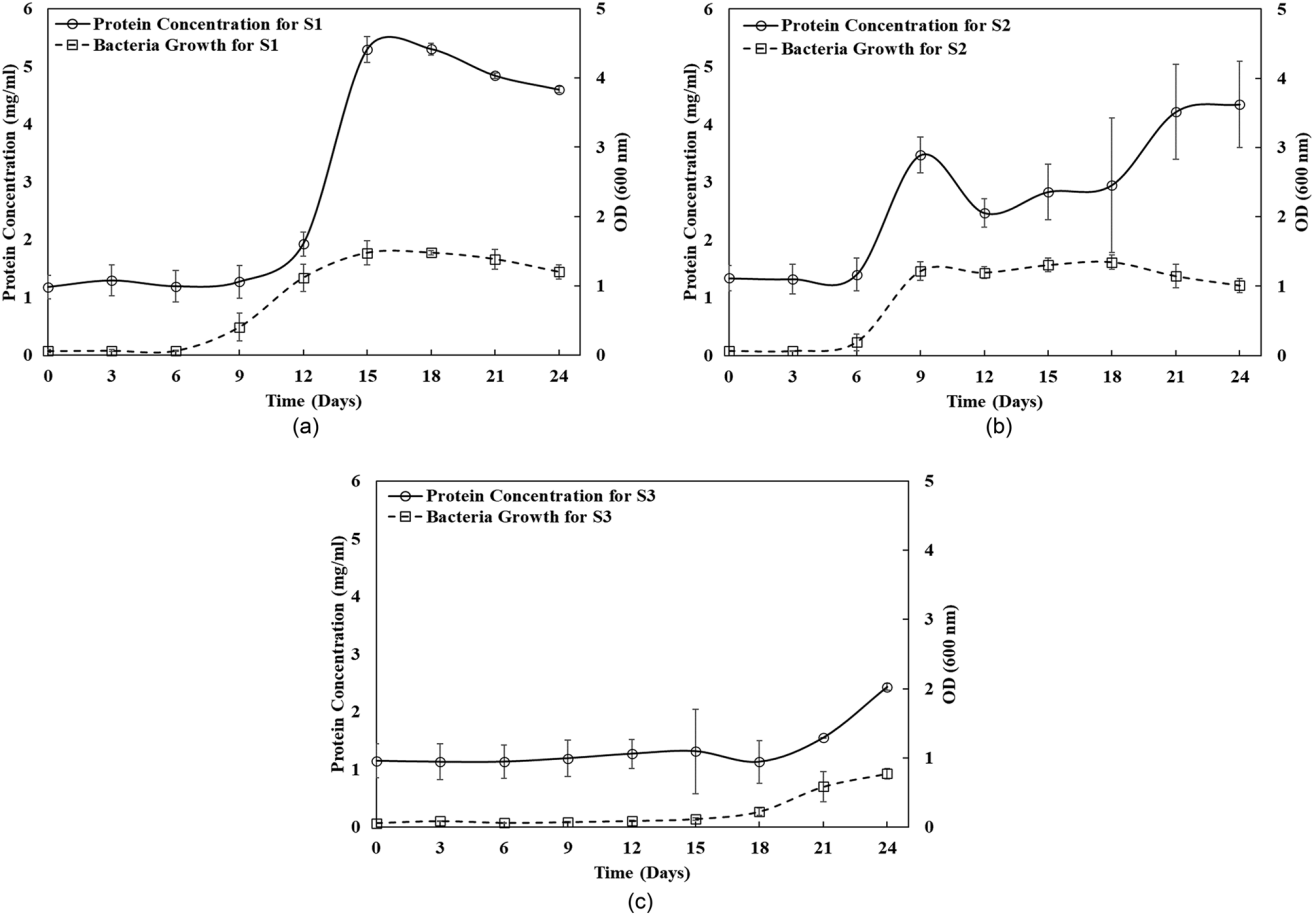
Growth phase of psychrophilic microbes (**a**) *Sporosarcina psychrophile* strain S1, (**b**) *Sporosarcina globispora* strain S2, and (**c**) *Polaromonas hydrogenivorans* strain S3 at a temperature of 4 °C for 24 days and the concentration of released protein in the extracellular solution measured at an interval of three days. For this study, the pH of the microbial growth was maintained at neutral (pH 7), their optimum pH (data not shown). The maximum, minimum and average calculated standard deviations (σ) for protein concentration data set are σ_max_ = 0.83, σ_min_ = 0.00 and σ_avg_ = 0.33 and for bacteria growth data set are σ_max_ = 0.22, σ_min_ = 0.00 and σ_avg_ = 0.08, respectively.

Figure 1 presents the growth profile of the three psychrophilic microbes, *Sporosarcina psychrophile* strain (S1), *Sporosarcina globispora* strain (S2)*, and Polaromonas hydrogenivorans* strain (S3)*,* selected for this study, and their released protein concentration in the extracellular growth fractions after a regular interval of 3 days. As witnessed in Fig. 1a–c, cultures S1 and S2 grew faster compared to S3 to enter their exponential growth phase from the lag phase by day 6. In the case of S3, this phase shift only occurred on the 16th day of incubation. On a comparative note, S1 growth based on OD 600 nm readings peaked on the 15th day, continued to the stationary phase till the 18th day, and immediately moved to the slow death phase. In the case of S2, peak growth was witnessed on the 9th day, and the stationary phase continued from 15th to the 18th day. After that, S2 bacteria shifted to the death phase. Interestingly, S3, under the same condition, started to grow exponentially only on the 15th day. Based on the results described in the later section, the growth phase was decided to continue for 24 days. In this period, S3 continued its exponential growth and showed no stationary phase.

Simultaneously, at a similar interval of growth phase monitoring, the extracellular supernatant was collected and tested to determine any effect on the thermal changes in porous media. We hypothesized, expected, and tested here that the microbes, while being grown under the cold temperature of 4 °C, would release ice crystal arresting proteins. It is important to note that the initial protein concentrations reflected in Fig. 1, until 6th day, should be the background proteins in the media. As all 3 strains were in their lag phase till then, we do not expect them to produce or release any psychrophilic proteins. Therefore, any effect of the solution on ice crystallization is understandably not validated until the 6th day. During the experimentations, the maximum protein concentrations in the extracellular supernatant were measured for strains S1 (5.3 mg/ml) on Day 15, S2 (4.35 mg/ml) on Day 21, and S3 (2.43 mg/ml) on Day 24. Interestingly, both S1 and S3 strains exhibited the peak levels of maximum proteins in their culture supernatant during the late log or early stationary growth phase. In contrast, S2 released its maximum proteins during the death phase.

### Thermal properties-based results

**Figure 2:**
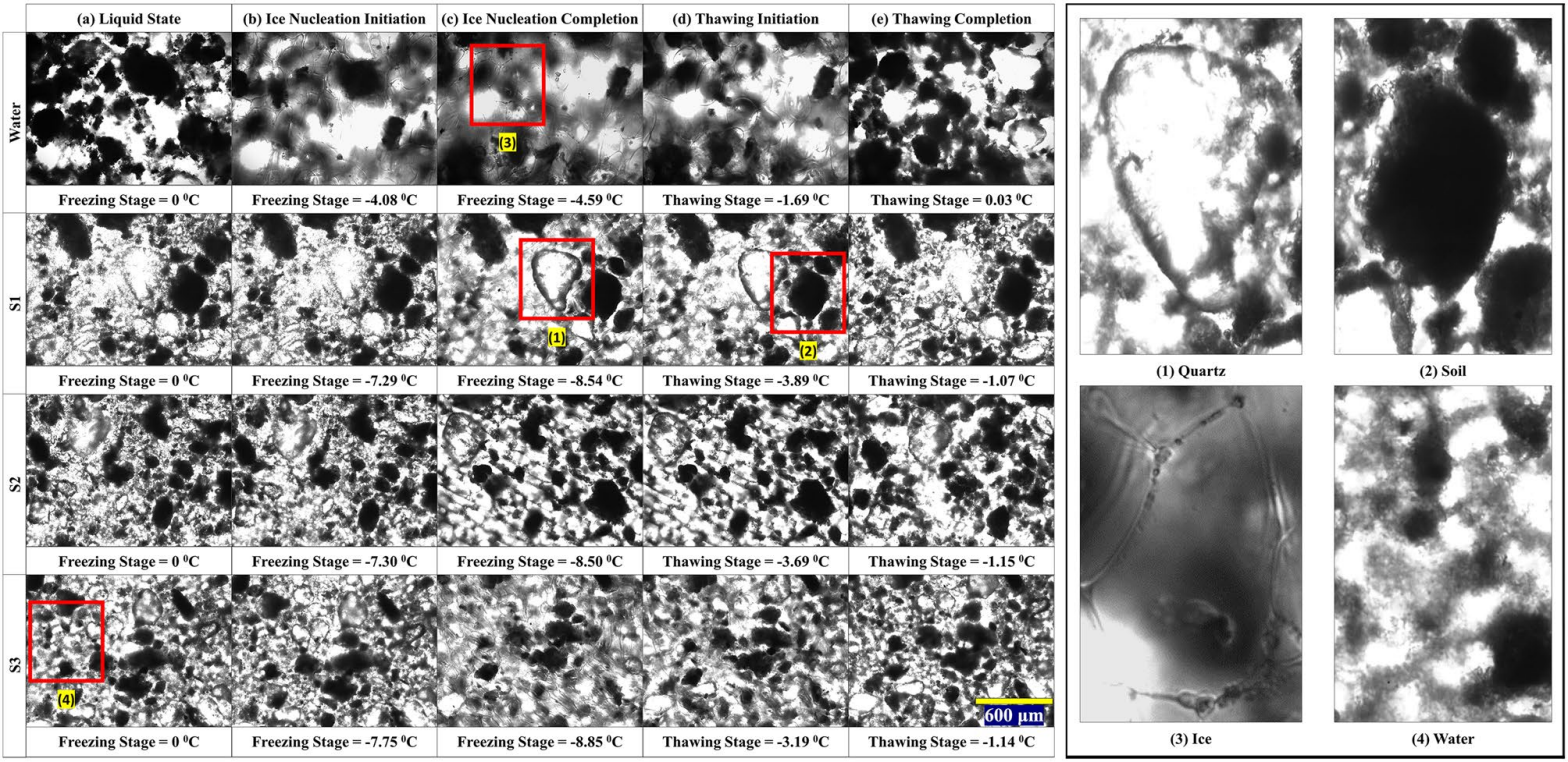
(**a**) to (**e**) showing phase changing steps of soil mixed with porous media (water) and supernatants under freezing and thawing procedure for water, S1, S2, and S3. The magnified images on the right most define quartz, soil, ice crystal, and water, as marked as 1, 2, 3, and 4 in the principal images.

In this phase of the experiment, 20 µL of supernatants collected on days 6, 9, 12, 15, 18, 21, and 24 from actively growing cultures of S1, S2, and S3 psychrophiles were combined with 10 mg of soil grains. Subsequently, thermal characteristics during freezing and thawing were examined. Figure 2 illustrates these thermal results alongside microscope images capturing the phase changes (Fig. 2a–e) in porous media, depicting the transitions from liquid to ice crystal and vice versa.

During the freezing stage, at 0 °C temperature, the water (control), S1, S2, and S3 did not change their physical state (liquid to frozen), as seen in Fig. 2a. As the temperatures decrease below 0 °C, the ice formation (Fig. 2b) initiated on the specimen surface (i.e., − 4.08 °C for water-soil mixture), which will be referred to as ‘Ice Nucleation Initiation’. In Fig. 2c, these ice formations start to take stable forms with polygonal shapes at much lower negative temperatures (i.e., − 4.59 °C for water-soil mixture). After this point, no new crystal formation was observed, and this signifies the end of the freezing stage referred to as ‘Ice Nucleation Completion’. In Fig. 2d, the frozen crystals started to melt (i.e., − 1.69 °C for water-soil mixture), which is referred to as ‘Thawing Initiation’. At this negative but higher temperature than the freezing point temperature, the ice crystals spontaneously melt and move from place to place. This signifies the potential increment of Gibbs free energy to melt the ice crystals by increasing enthalpy and entropy. Finally, Fig. 2e indicates the end of the thawing point (i.e., 0.03 °C for water-soil mixture) where no ice crystal is visible for any water or supernatants mixed soil. This will be called the ‘Thawing Completion’ step.

**Figure 3:**
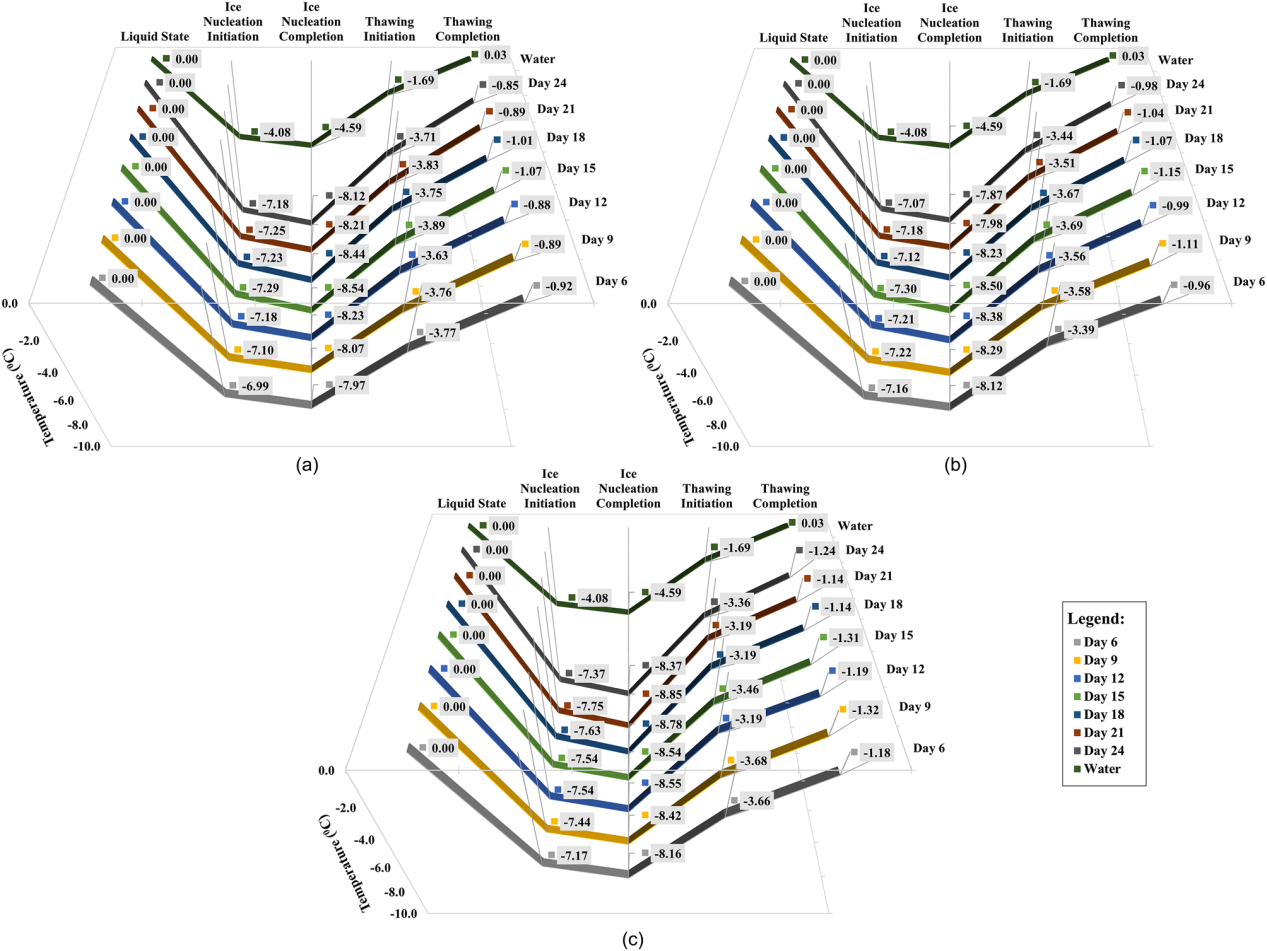
Phases changes and succeeding temperature of soil mixed with (**a**) S1, (**b**) S2, and (**c**) S3 psychrophilic microbes’ extracellular solution (test samples) on further growth. The vertical axis on the left side of the figure shows the corresponding temperature for the phase changes. The horizontal axis shows all the steps of the phase changes, and the Z-axis shows the resultant days of the experiment. The maximum, minimum and average calculated standard deviations (σ) for this data set are σ_max_ = 0.13, σ_min_ = 0.01 and σ_avg_ = 0.06, respectively.

Figure 3 provides a detailed account of the temperatures attributable to the phase change steps pictured in Fig. 2 as a crucial means for further understanding the thermal characteristics properties of the extracellular microbial solutions used for arresting ice crystallization of porous media in soil. The ice nucleation initiation stage illustrated in Fig. 2 represents the start of ice formation. This initiation was recorded at − 4.08 °C for the control sample (no psychrophile, just soil–water), but for the soils mixed with supernatants (S1, S2, and S3), the ice nucleation initiation (T_ini_) transpired at a comparatively lower temperature. As shown in Fig. 3a, for psychrophile S1, T_ini_ was recorded at − 6.99 °C on day 6 sample, where the microbe was still in its lag phase of growth (Fig. 1). The lowest T_ini_ for S1 treated samples was observed on day 15, at − 7.29 °C, which was 3.21 °C lower than the control. The depression recorded for T_ini_ between day 6 and 15 reads minimal, but still, it is significant to believe that the S1 is active at lower temperatures depicting antifreeze activities. For thawing initiation (T_ti_), the control sample started to thaw at − 1.69 °C; for samples of S1-soil mixtures, ice thawing was recorded with T_ti_ being in the range of − 3.63 °C on day 12, and − 3.89 °C on day 15. To note, for microbial culture S1, day 15 also marks the time when a maximum of the protein concentrations was recorded in the extracellular solution of culture. The phase change diagram for S2 samples mixed soil samples over time of culture growth is shown in Fig. 3b. For S2 samples, the lowest T_ini_ and T_ti_ were recorded for day 15, with values of − 7.30 °C and − 3.69 °C, respectively. Likewise, for Sample S1, the closest initiation temperature for both steps were observed for day 6 samples, the period triggering S1’s move from its lag phase to the exponential growth phase. For S3 samples’ the phase change diagram over its growth phases is shown in Fig. 3c. The lowest T_ini_ and T_ti_ can be seen registered with day 21 and 9 with values of − 7.75 °C and − 3.68 °C, respectively. All three S1-S3 cultures depicted analogous freezing and thawing steps initiation temperature, depicting antifreeze activities of psychrophilic microbes.

#### Freezing point (T_f_)

In the ice nucleation completion step, the freezing point temperatures were observed for water, S1, S2, and S3 supernatant mixed with soil in Fig. 3. Here, the control test conducted using 20 µL water mixed with 10 mg silt showed an average freezing temperature of − 4.59 °C. On the other hand, the same amount of soil combined with 20 µL bacterial extracellular solutions displayed depressions in the freezing temperatures, as shown in Fig. 3, with the depression correlating directly with the increment in the protein concentrations for different test days. With sample S1, the most significant freezing point depression was recorded for day 15 samples, where a freezing point of − 8.54 °C was observed with respective protein content in the culture solution measured at 5.30 mg/ml. Subsequently, the freezing point depressions started to decrease, coinciding with the decrement of protein concentrations in the culture solution. Like the S1 culture solution, the lowest freezing point of − 8.50 °C was observed for S2, on day 15, with protein content at 2.83 mg/ml. For the S3 sample culture supernatants, the lowest freezing point of − 8.85 °C was observed on day 21, with the measured protein concentration in the sample being 1.55 mg/ml. Amongst the three psychrophilic strains tested, overall, S3 seemed to comprehend better control over the freezing point depression compared to microbes S1 and S2. It is to be noted that initially, the growth medium exhibited a freezing point of − 6.19 °C on day zero. Over the course of days 0 to 24, the medium's impact diminished to − 5.88 to − 5.01 °C, suggesting a decreasing influence of chemicals. Conversely, the psychrophilic bacteria-treated soils showed an increasing protein concentration over time, implying that chemicals may have minimal influence on our treatment outcomes.

#### Thawing point (T_t_)

The thawing completion temperatures in Fig. 3 represent a complete unfrozen state of the control and bio-treated (S1, S2, S3 with soils). The control sample consisting of frozen water-soil thawed at 0.03 °C. However, the bio-treated soils (S1, S2, S3 with soils) thawed at negative temperatures. The thawing temperature for the S1-soil was between the range of − 0.85 to − 1.07 °C on days 15 and 24 respectively. Like S1, the thawing temperature for S2 treated soils was between the range of − 0.96 to − 1.15 °C, where the lowest temperature was observed on day 15 while the highest was observed on day 6. For S3, the thawing temperature was between − 1.14 to − 1.32 °C, where the lowest temperature was observed on day 9, and the highest was observed on days 18 and 21.

#### Freezing point depression difference (ΔT_f_)

**Figure 4:**
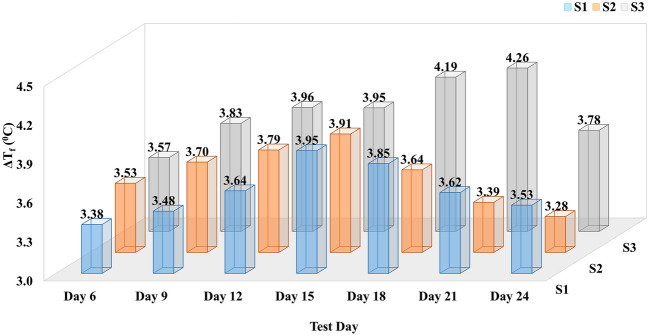
Bar diagram demonstrating the difference between the freezing point depression of soil mixed with water and three psychrophilic microbes’ (S1, S2, and S3) extracellular solution (test samples) on different growth days. Here, the X-axis shows the resultant days of the experiment, the Y-axis presents the freezing point depression difference (ΔT_f_) in degree Celsius temperature, and the Z-axis shows three psychrophilic microbes IDs. The maximum, minimum and average calculated standard deviations (σ) for this data set are σ_max_ = 0.11, σ_min_ = 0.06 and σ_avg_ = 0.09, respectively.

Figure 4 presents the difference in the freezing point depression (ΔT_f_) between soil mixed with water and proteins over different days of the growth period by using Eq. (1). For S1 and S2, the maximum freezing point depression difference was observed on Day 15 with ΔT_f_ = 3.95 °C and ΔT_f_ = 3.91 °C, equivalent to depression of 85.98% and 85.26%, compared to control soil with water, respectively. The lowest depression difference observed for them is on Day 6 and Day 24, respectively, which is still ΔT_f_ > 3 °C. A significant difference in performance was marked for S3 with ΔT_f_ = 4.26 °C on Day 21 with 92.81% depression. Even the lowest ΔT_f_ = 3.57 °C was found on Day 6 when the growth of bacteria or concentration of ice crystal inhibiting protein was minimal. The results conclude that some extracellular products secreted by the microbes lowered the porous media's freezing point and showed significant performance.

#### Thermal hysteresis (TH) activity

**Figure 5:**
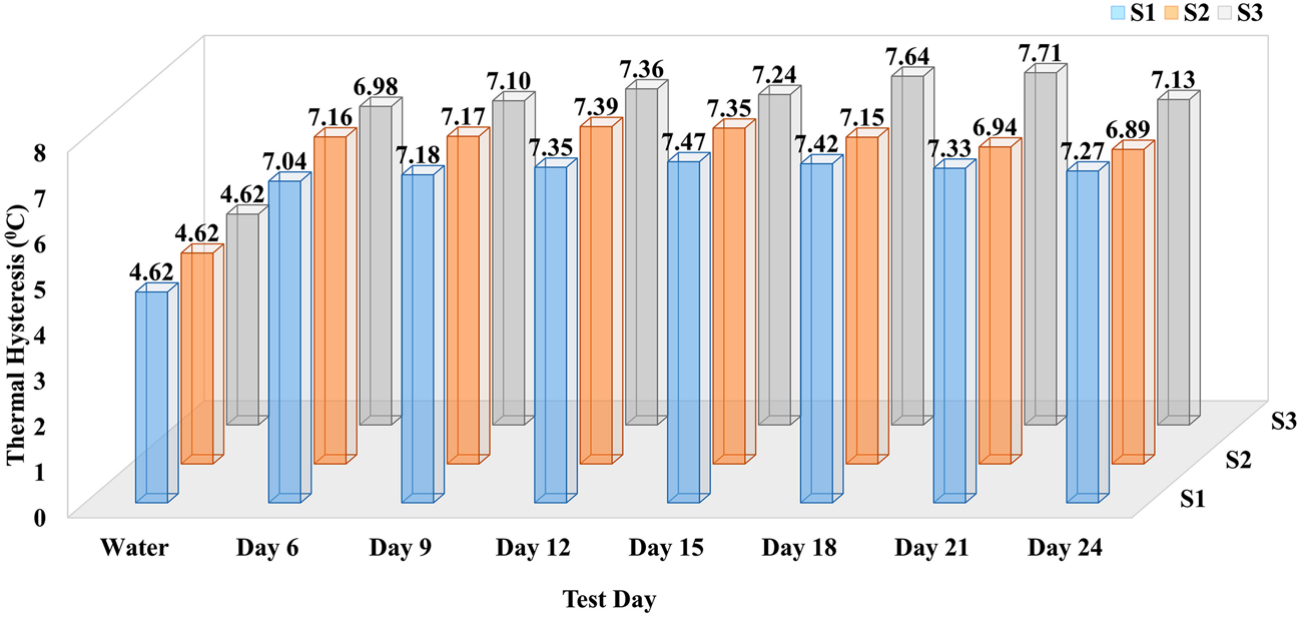
Comparative presentation of thermal hysteresis activity of soil mixed with water (control) and three psychrophilic microbes’ (S1, S2, and S3) extracellular solution (test samples) on different growth days. Here, the X-axis shows the resultant days of the experiment, the Y-axis presents thermal hysteresis in degrees Celsius temperature, and the Z-axis shows three psychrophilic microbes ID. The maximum, minimum and average calculated standard deviations (σ) for this data set are σ_max_ = 0.16, σ_min_ = 0.03 and σ_avg_ = 0.08, respectively.

The effect of three psychrophilic microbes’ (S1, S2, and S3) extracellular solution on enhancing the thermal properties of frost-susceptible soils can be described more accurately with Thermal hysteresis (TH) activity. The TH is the thermal gap between freezing and thawing points which were recorded and calculated using Eq. (2). Its dependability on the tested samples is shown in Fig. 5. As expected, the lowest 4.62 °C TH activity was observed for the water-soil mixture. Compared, all the extracellular protein-treated samples registered higher TH activity. The TH activity for S1 ranged from 7.04 to 7.47 °C for different growth periods, with the maximum observed on Day 15. This day of maximum TH recorded for the bacterial sample S1 was due the highest protein content recorded in its extracellular solution, i.e., 15th day for the S1 culture growth at 4 °C (Fig. 1). For S2, the maximum TH activity of 7.39 °C was also observed on Day 12, and the lowest activity was 6.89 °C on Day 24. For culture S3, the maximum TH activity of 7.71 °C (also the highest activity recorded among S1, S2, and S3) was seen on Day 21, and the lowest activity was 6.98 °C on Day 6.

### Delayed freezing and premature thawing analysis

**Figure 6:**
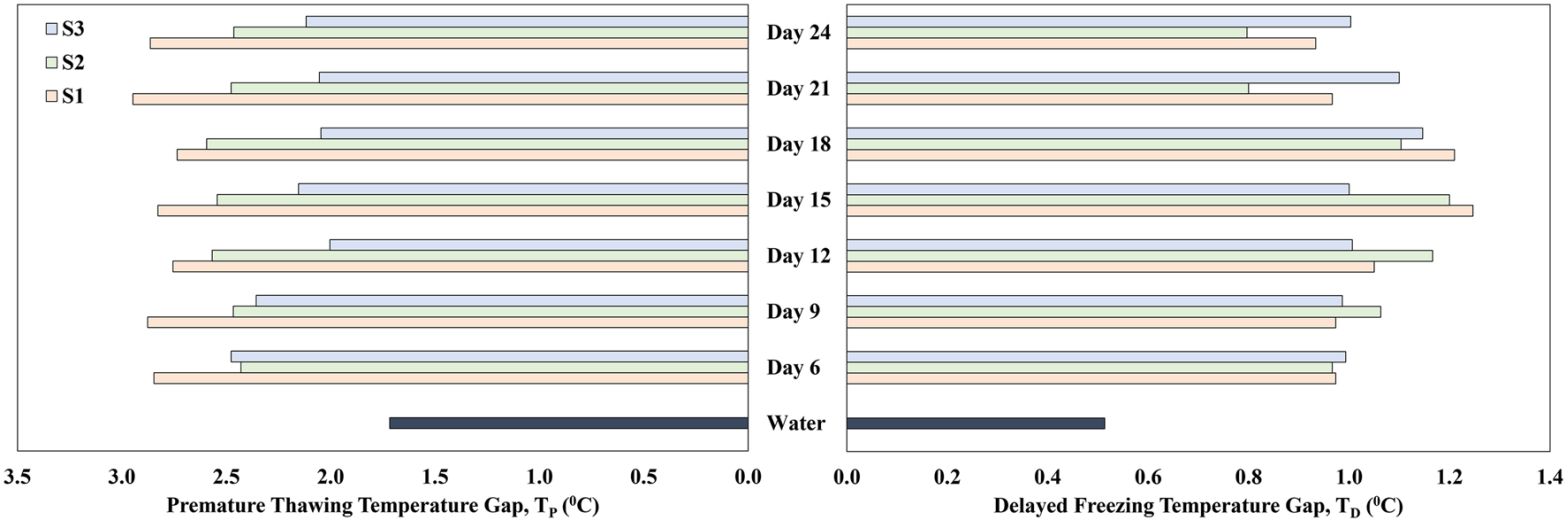
Premature Thawing (T_P_) and Delayed Freezing (T_D_) Analysis / Comparative thermal gap between ice nucleation initiation and completion soil mixed with water and psychrophilic microbes’ (S1, S2, and S3) extracellular solution (test samples) on further growth. The maximum, minimum and average calculated standard deviations (σ) for this data set are σ_max_ = 0.14, σ_min_ = 0.04 and σ_avg_ = 0.08, respectively.

In the freezing point results, we have explained how AFP molecules adhere to the crystal's surface and inhibit ice crystal development. Thus, the freezing point of porous media is lowered to a new freezing point by preventing the creation of ice crystals. This is also true for the lower ice nucleation initiation temperature. Here, we quantify this observation with ‘Delayed Freezing (T_D_),’ where the difference between the ice nucleation initiation (T_ini_) and ice nucleation completion (T_inc_) have been calculated by using Eq. (3). From Fig. 6, where the horizontal axis shows the delayed freezing, the observations for the maximum delay in the freezing temperature for sample S1 can be seen for the day 15th with a value of 1.25 °C when the microbial culture S1 had a maximum of the protein concentrations recorded in the extracellular solution of culture. Comparatively, the water soil control had a T_D_ of only 0.51 °C, which is a much faster process for freezing to complete. For culture S2, the maximum T_D_ temperature of 1.20 °C was found for Day 15, whereas the maximum T_D_ temperature for culture S3 was 1.15 °C on Day 18. Notably, for S1, S2, and S3-mixed soil, the lowest freezing point and maximum or secondary maximum delayed freezing value were observed on the same day, which coincides with the knowledge that the AFPs control ice crystal growth to a much lower freezing point.

Similar to the delayed freezing, for the manuscript’s reference, the difference between the thawing initiation and thawing completion has been termed ‘Premature Thawing (T_P_)’ and has been calculated using Eq. (4). The water-soil control recorded a thermal gap of 1.72 °C between its thawing initiation at − 1.69 °C and thawing completion at 0.03 °C (Fig. 6). Conversely, the culture test samples have higher Premature Thawing. For culture S1, each sample from a different growth phase showed a T_P_ of 2.75 °C or above. The premature thawing characteristic for S2 showed a T_P_ of 2.43 °C or above; for S3, the T_P_ was recorded at 2 °C or above.

## Discussion

This study is the first attempt to evaluate the ability of psychrophilic bacteria to inhibit the ice growth in frost-susceptible soil. Earlier studies have shown that AFP can lower the freezing point of water as well as increase the thermal hysteresis of the solution^16^. In our study, we demonstrated that secreted proteins from three selected psychrophilic bacteria have the ability to lower the natural freezing point of porous media and soil composite from − 4.59 °C to − 8.54 °C which matches with earlier studies. The possible mechanisms can be attributed to the AFP molecules adhering to the ice crystal’s surface and generating "kinks" or "defects" resulting in inhibition of ice crystal initiation and depression^15,39^. The varying peak means of such depression is also found to be dependent on not only the concentration of the released proteins but also depends on the specific types of proteins that have been secreted by the individual proteins^40,41^. Without precise identification, establishing a direct correlation between protein concentration and performance is challenging, emphasizing the need for further analysis. Nevertheless, the quantitative number of depressions for the protein solutions compared to the control specimen (ΔT_f_) is significantly superior (maximum witnessed 4.26 °C for S3) which make the bacteria ideal candidates to use in field to naturally control the ice growth to a much lower freezing temperature.

We have found maximum TH activity of 7.71 °C for our S3 protein mixed soil composite. Previously, the increase in the hysteresis activity of the water had been reported for antifreeze proteins (AFPs) from varied sources, where the thermal gap was much more significant than the TH of water alone^42,43^. When water and an antifreeze protein are combined, the antifreeze protein attaches to the ice crystals and stops their growth. The structure of the ice-water contact is altered as a result of this binding interaction. Adsorbing on the ice crystals' surface, the antifreeze protein molecules prevent additional water molecules from joining the crystal lattice. As a result, ice crystals are prevented from forming and water is able to stay liquid at a temperature below its normal freezing. Such results for treated soil composite made us comprehend that the extracellular solutions of the three psychrophilic microbes under study are carrying AFP or its analogous protein produced and secreted by the microbes.

Beside TH activity, from the delayed freezing (T_D_) analysis, we have identified the porous media mixed soil freezes much faster (0.51 °C) where the proteins reduce the growth of ice crystallization of soil composite (maximum seen 1.25 °C for S1) at a fixed temperature ramp. This signifies the availability of higher Gibbs free energy created by the proteins to prevent ice crystallization from ensuing at shorter temperature gaps^17^. In all three cases, as the protein concentration was recorded to increase in the extracellular solution, the delaying of freezing was a more distinct phenomenon. Similarly, from premature thawing (T_P_) analysis, we have also similar phenomena taking place where porous media mixed soil thaws at a gap of 1.72 °C where minimum temperature gap required for the proteins treated soil was 2 °C or above. This quantified result shows that AFPs in the extracellular solutions can generate enough Gibbs free energy to dissipate the ice crystal at a negative temperature. Our experiment also shows the protein-treated soil composite thawed at a negative temperature. As the variable in this experiment was only the extracellular growth solution of the three psychrophilic microbes, we apprehend that a component of the growth medium or the extracellular product secreted by the microbes as well as the soil itself have a role to play in initiating thawing at a much lower temperature by their inherent properties. However, this was kept beyond the scope of this study.

## Conclusion

In summary, the present study created a baseline to inhibit the ice growth in frost-susceptible soil using the extracellular proteins of psychrophilic bacteria. Soil mixed with porous media or water showed freezing point of − 4.59 °C, thawing point of 0.03 °C and thermal hysteresis 4.62 °C. The results clearly suggested that the protein solutions from the psychrophilic bacteria led to increased freezing point depression, where strain S3 demonstrated the strongest control over freezing point depression in soil samples, with the lowest observed freezing point of − 8.85 °C on day 21 and a protein concentration of 1.55 mg/ml. All the bio-treated soils exhibited thawing completion temperatures below 0 °C, with S3 showing the lowest temperature range of − 1.14 to − 1.32 °C. The extracellular solutions of psychrophilic microbes significantly increased the thermal hysteresis (TH) activity in frost-susceptible soils compared to the water-soil mixture, with TH values ranging from 7.04 to 7.71 °C. These findings indicate that the protein solutions may contain IBPs such as AFPs or CSPs, or both which play a role in influencing the freezing and thawing behavior of the soil, potentially through their ability to modify the properties of water and ice. These psychrophilic IBPs, in the future, should be characterized.

## Materials and methods

### Frost-susceptible porous soil

According to Federal Highway Administration, a very high frost-susceptible soil, homogeneous silt, was selected for this study to overcome the complexity of soil properties^44^. Earlier thermal studies on silt mixed with a variable amount of water content have exhibited supercooling state temperatures around − 6 to − 4 °C with a meager amount of unfrozen water content^45,46^. The pH of the soil is 7.12 at a moisture content of 100%. The mineral content of the fine-grained soils was determined using X-ray diffraction (XRD), where 51.39% of Quartz and 31.78% of Albite were found to dominate minerals.

### Psychrophilic strains

For this study, three psychrophilic strains, *Sporosarcina psychrophile* strain 1530 (Strain 1), *Sporosarcina globispora* strain 3396 (Strain 2), and *Polaromonas hydrogenivorans* strain 41369 (Strain 3), were procured from the ARS (NRRL) culture collection center (Peoria, IL).

### Growing bacteria and collecting enzymes

All three strains were maintained on Luria Broth agar plates (Sigma-Aldrich, St Louis, MO) at 4 °C. The inoculums were prepared by transferring a colony from each plate into 5 ml Luria broth in 15 ml culture tubes and followed by incubation for ten days at 4 °C, under aeration with rotation at 150 rpm in a Solaris™ Open Air Orbital Shaker (Thermo Fischer Scientific, USA). The production cultures were then prepared by inoculating 2% (v/v) ml of inoculums into 25 ml of the sterilized production medium in 100 ml conical Erlenmeyer flasks under identical conditions until the desired growth phase (exponential or stationary) was obtained. The production medium was composed of casein peptone (pancreatic) (17 g/L), dipotassium hydrogen phosphate (2.5 g/L), glucose (2.5 g/L), sodium chloride (5 g/L), and soya peptone (papain digest.) (3 g/L). The pH of the medium was adjusted to 6.8 by adding hydrochloric acid (Sigma-Aldrich). At periodic intervals of 6, 9, 12, 15, 18, 21, and 24 days, 2 ml of the culture samples were withdrawn from the growing cultures. The growth phases of the microbial cultures were measured from the optical absorbance at 600 nm (A600) using an Epoch Microplate Spectrophotometer (Agilent BioTek, CA, USA). The cells were then harvested by centrifuging the cultures at 4 °C at 10,000 rpm for 5 min. The cold shock proteins mixed enzymes in the culture supernatant produced and secreted by the cold-induced bacterial cells were mixed with soil for thermal testing. The estimation of protein concentration was carried out using a Pierce™ Coomassie (Bradford) Protein Assay Kit following the manufacturer’s protocols.

### Investigation procedure of ice crystallization in porous media

**Figure 7:**
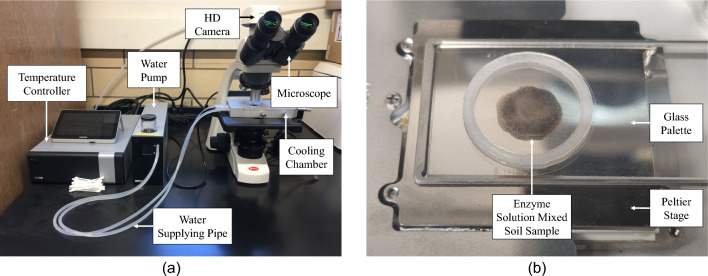
Test setup.

A thermoelectrically controlled cooling device (Linkam’s LTS120) was used in this study to measure the freezing and thawing point of the psychrophilic bacteria-secreted protein-treated soil. This cooling device has a controller to set the rate of temperature, a cooling chamber, a water pump to carry the heat energy out of the cooling chamber, and a microscope with a camera to observe and record the phase changes of the testing specimen. A Peltier cooling stage is placed inside the chamber of the cooling device. This cooling chamber can depress temperatures from 120 to − 40 °C, and it can be controlled with a precision of ± 0.01 °C. To observe the test specimens’ phase changes inside the cooling device, a brightfield microscope was equipped with 20 × long working distance objectives. Figure 7a shows the setup of the cooling device. Three sets of slurry samples were prepared by adding 20 μL of water or protein solution with 10 mg of silt soil and kept at 4 °C temperature for 24 h so that the proteins could aptly mix with the soil grains. After preparing the testing samples, a small amount of specimen from the 20 μL samples was placed on the Peltier stage inside the cooling device. A temperature ramp of 0.5 °C was maintained to freeze and thaw the samples. A digital camera recorded the full microscopic test from time zero to the final temperature-reaching time. For further analysis, all the recorded images were processed and inspected by taking inspiration from a biotechnology articel^47^. Figure 7b shows the typical testing specimen placed on the glass palette of the Peltier stage.

Table 1 shows the test strategy used in this investigation to determine how psychrophilic proteins affect freezing point depression. 20 μL of the respective protein solutions collected on days 3, 6, 9, 12, 15, 18, 21, and 24, as well as 20 μL of water as control, have been mixed with 10 mg of silt and tested.

**Table 1:** Summary of test design.

Mixed solution	Strain ID	Silt (mg)	Protein solution (μL)	Added water (μL)
Porous media (water)	–	10	–	20
*Sporosarcina psychrophila*	S1	10	20	–
*Sporosarcina globispora*	S2	10	20	–
*Polaromonas hydrogenivorans*	S3	10	20	–

### Parameters studied and formulas used in this study

In this study, we have investigated the freezing point (T_f_) and thawing point (T_t_) of the water and protein solution mixed soil. Further, from the experimented data, we calculated thermal hysteresis activity (TH) using Eq. (2). The freezing point depression difference (ΔT_f_) between the control and the protein-treated soil was received using Eq. (1). Based on the significant shifting of ice nucleation initiation (T_ini_) and ice nucleation completion (T_inc_) as well as the thawing initiation (T_ti_) and thawing completion (T_tc_), we have measured their differences by using Eqs. (3) and (4), respectively. We have termed them as ‘Delayed Freezing (T_D_)’ and ‘Premature Thawing (T_P_)’.

1 $${\Delta T}_{{\text{f}}} = {\text{Freezing}}\;{\text{Point}}\;{\text{of}}\;{\text{Protein}}\;{\text{Treated}}\;{\text{Soil }}{-}{\text{Freezing}}\;{\text{Point}}\;{\text{of}}\;{\text{Water}}\;{\text{Treated}}\;{\text{Soil}}$$

2 $${\text{TH}} = {\text{Thawing}}\;{\text{Point}}\;{\text{of}}\;{\text{Treated}}\;{\text{Soil}}{-}{\text{Freezing}}\;{\text{Point}}\;{\text{of}}\;{\text{Treated}}\;{\text{Soil}}$$

3 $${\text{T}}_{{\text{D}}} = {\text{Ice}}\;{\text{Nucleation}}\;{\text{Completion}}\;{\text{Temperature}}{-}{\text{Ice}}\;{\text{Nucleation}}\;{\text{Initiation}}\;{\text{Temperature}}$$

4 $${\text{T}}_{{\text{P}}} = {\text{Thawing}}\;{\text{Completion}}\;{\text{Temperature}}{-}{\text{Thawing}}\;{\text{Initiation}}\;{\text{Temperature}}$$

### Statistical analysis performed

Unless otherwise mentioned, all the experiments were performed in triplicate, and data were averaged and presented as the mean ± standard deviation (SD).

## Data Availability

The data used to support the findings of this research are included within the paper.
